# The waves that make the pattern: a review on acoustic manipulation in biomedical research

**DOI:** 10.1016/j.mtbio.2021.100110

**Published:** 2021-03-24

**Authors:** A.G. Guex, N. Di Marzio, D. Eglin, M. Alini, T. Serra

**Affiliations:** a AO Research Institute Davos, Clavadelerstrasse 8, 7270 Davos, Switzerland; b Department of Health Sciences, Università del Piemonte Orientale (UPO), Novara, Italy

**Keywords:** Sound, Biofabrication, Pattern, Standing waves, Faraday waves

## Abstract

Novel approaches, combining technology, biomaterial design, and cutting-edge cell culture, have been increasingly considered to advance the field of tissue engineering and regenerative medicine. Within this context, acoustic manipulation to remotely control spatial cellular organization within a carrier matrix has arisen as a particularly promising method during the last decade. Acoustic or sound-induced manipulation takes advantage of hydrodynamic forces exerted on systems of particles within a liquid medium by standing waves. Inorganic or organic particles, cells, or organoids assemble within the nodes of the standing wave, creating distinct patterns in response to the applied frequency and amplitude. Acoustic manipulation has advanced from micro- or nanoparticle arrangement in 2D to the assembly of multiple cell types or organoids into highly complex in vitro tissues. In this review, we discuss the past research achievements in the field of acoustic manipulation with particular emphasis on biomedical application. We survey microfluidic, open chamber, and high throughput devices for their applicability to arrange non-living and living units in buffer or hydrogels. We also investigate the challenges arising from different methods, and their prospects to gain a deeper understanding of in vitro tissue formation and application in the field of biomedical engineering.

## Highlights

• Work on sound waves to spatially control particulate systems is reviewed.
• Classification of surface acoustic waves, bulk acoustic waves, and Faraday waves.
• Sound can be used to arrange, separate, or filter polymer particles.
• Sound can pattern cells in 3D to induce morphogenesis.
• Long-term applied sound induces differentiation and tissue formation.

## Graphical abstract

**Figure:**
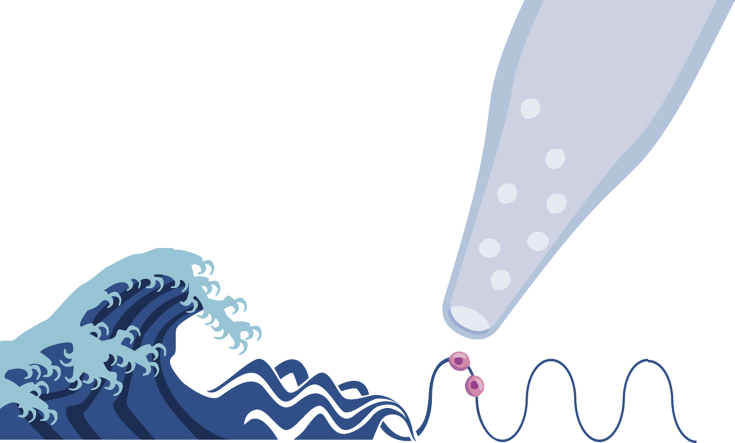

## 1 Introduction and outline

Since its first mentioning in 1993 by Langer and Vacanti [1], tissue engineering has evolved from a laboratory-based subject of research to some impactful therapeutic solutions. A plethora of efforts in countless research laboratories have contributed to the development of new culture conditions, materials, devices, and strategies, that are reflected in an ever-increasing number of records in the literature. To date, however, the number of products that have been successfully translated to the clinics lies in stark contrast to the sky-rocketing number of published studies [[2], [3], [4], [5]]. Among many other hurdles on the path to clinical application, mimicking the sheer complexity of natural tissue with respect to architectural organization, high cell density, biochemical constitution, and biophysical cues ​remains a major obstacle. Cells are responding to their physicochemical environment and, in response, adapt their phenotype but also constantly remodel their extracellular matrix (ECM). This dynamic, three-dimensional (3D) environment and the symbiotic relationship between ECM and cells of natural tissues can only marginally be reproduced in vitro. Addressing this need by new methods and sophisticated approaches would significantly contribute to the success of in vitro models and will be key to their translation to the clinic. Historically, achieving high cell density, mesenchymal condensation for chondrogenesis, and 3D constructs to push in vitro organ formation has primarily been addressed by the development of organoids or spheroids [6], which are most well known in the field of cancer research [7] and are achieved by, for example, hanging drop methods, within low adhesion plates or via bioprinting**.** These techniques have a lot of merit, but require a lot of technical expertise that comes with some drawbacks on the reproducibility and can only insufficiently provide additional cues from material properties. The combination of fine-tuned material properties and cutting-edge technologies have tremendously moved the field forward, culminating in a discipline now referred to as *Biofabrication* whose advantages, disadvantages, and promises have been reviewed in length [[8], [9], [10]]. Biofabrication strategies are manifold and encompass a plethora of methods. Common to all methods is the desire to combine material approaches and cell seeding strategies to achieve complex 3D tissues. Among them, the concept of using different molecular building blocks to address tissue complexity in a bottom-up approach has gained increasing interest, owing to its advantages compared to conventional top-down bioprinting approaches [11,12]. Homogeneous seeding at a density that comes close to the native one, controlled spatial distribution of cells within a construct, and essentially no limits for scaling-up are only a few of their thrilling benefits. In addition, building blocks are formed from non-living and living materials of different sizes such as single cells, spheroids, or cell sheets. Independent of the many different techniques, instruments, and approaches, there seems to be at least some consensus on the following points: (i) the synthetic material must provide an environment for cells to adhere, proliferate, and/or differentiate; (ii) spatial cell patterning and cell condensation to mimic the high native cell-density in tissues have been appreciated as a vital prerequisite for successful in vitro tissue engineering; and (iii) achieving high cell density within a material must be accomplished without the expense of producing heterogeneous constructs and ideally by remote, cell friendly manipulation. A particularly interesting aspect in this regard is the remote assembly of building blocks and external control over the spatial organization of individual cells, cell aggregates, or spheroids within a carrier matrix. Contact-less, non-destructive manipulation is particularly cell-friendly and has been explored by use of optical, magnetic, or acoustic fields [13]. The latter one takes advantage of acoustic waves and their generated radiation or hydrodynamic forces that act on cells. By this, externally applied sound waves have the potential to manipulate cells within a liquid or hydrogel and position them in 3D by changing the applied frequency and amplitude. Acoustic manipulation captivates by its versatility to use a broad selection of hydrogel precursors, its high resolution to produce micron-sized structure, and its potential to work with low cell numbers to create micro-tissues of physiologically relevant cell density—three main aspects that are limited with conventional approaches. This review aims to provide an overview of the existing literature in this new and rapidly evolving field that has the potential to revolutionize approaches in biofabrication.

Observing, analyzing, and using sound waves have been of interest to the scientific community for many decades, if not centuries. Sound waves can travel through different media, including solid, liquid, and gaseous phases. Based on their immediate and observable effect, waves at the liquid–air interface were among the most prominently studied ones. For example, what we now know as Faraday waves is an observation that dates back to 1831 when Michael Faraday noted in ​his diary *‘Mercury on tin plate being vibrated in sunshine gave very beautiful effects of reflection’* [14]. Moving on, 200 years later, standing waves within liquids or solids at a large frequency spectrum (<100 ​kHZ to MHz) emerged as a promising method to manipulate cells. In response, several review manuscripts were published during the last few years, focusing on approaches to separate systems of particles by sound waves within microchannels [15], discussing technical and theoretical aspects of fluid phenomena that arise during acoustic manipulation [16], microfluidics, and acoustic waves for particle and cell manipulation [17,18] or acoustic tweezers and their application in the biomedical field [[19], [20], [21]]. The cited publications provide a very good overview of the technical and theoretical aspects of sound-induced manipulation and we would like to refer the reader to these articles for additional information on the underlying theory. Methods for separation of particles could find use in oil recovery and emulsion separation, or filtration of micro-algae. With respect to biomedical application, blood screening, cell perfusion, and harvesting were among the most studied, with erythrocyte separation being a popular model with application in blood filtration. A review by Barani et al. [22] discussed the application of sound waves in cell studies, covering cell separation and co-culture systems within microchannels. The versatility of the methods has been further underpinned in a review by Go et al. [23] who focused on the potential of sound waves and microchannels to be used as sensors, for example, to detect nitric oxide (NO) as an early sepsis marker, identify breast cancer cells, polymorphisms in enzymes, or capturing deoxyribonucleic acid. The authors concluded that the potential for cheap, low-power, simple devices is given and will certainly advance the field further. In 2018, Olofsson and colleagues published a review that discusses work focusing on sound manipulation at high frequencies, that is, by ultrasound (MHz) without addressing particle or cell manipulation at lower frequencies [24]. The most recent review in the field was published in 2020 by Mohanty et al. [25] and provides an excellent overview of some of the underlying theoretical aspects in acoustic manipulation and discusses interesting approaches related to acoustic tweezers. Importantly, the review also addresses work where active swimmers (microbots or self-propelling micro agents) were manipulated by acoustic tweezers or a combination of acoustic and magnetic fields.

**Fig. 1:**
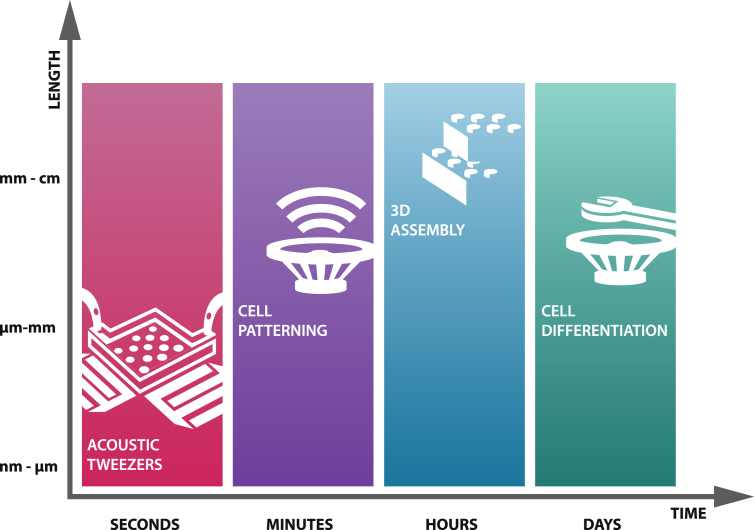
Use of acoustic manipulation on different length ​and time scales. Acoustic manipulation or sound patterning can be applied over seconds, minutes, or even hours or days to perform long-term stimulation. On the length scale, nano- or microparticles can be manipulated by acoustic tweezers, individual cells can be patterned within 2 or 2.5 dimensions, whereas 3D constructs were prepared by cell spheroid assembly.

In summary, acoustic manipulation has been largely studied in recent years. Both theoretical and experimental approaches on fluid mechanics and cell manipulation are reported, but often for a widely different scientific audience. In addition, the used devices and setups differ enormously among those reports, making it at times tricky for scientists new to the field to keep an overview. In this review, we aim to highlight the differences and similarities of the sound-inducing methodologies and strategies used and provide some insight on the underlying physics and wave theory. Furthermore, we will discuss boundary conditions with respect to material choice, biological considerations, and physicochemical parameters that are important to address before experimental execution and hope to provide an easy-to-understand summary of factors influencing successful sound-induced patterning. Different from the review papers already available, we will discuss work based on acoustic manipulation without the restriction to ultrasound or microchannels, but with major emphasis on the biological outcome and potential clinical application. The complexity in the field per se makes it difficult to categorize the published works. We, therefore, structure our review based on length ​and time scales, depending on the dimension of manipulated particles and resulting patterns, or depending on the duration of applied waves and the time needed to induce a particular cell response or pattern formation. These chapters are preceded by a summary of the theory. Fig. 1 schematically illustrates applications on the different time or length scales. A summary of the discussed research work is listed in Table 1.

**Table 1:** Summary of the cited research work.

Single object manipulation and assembly in 1D			
Concept	Device	Particle/cell type	References
Object trapping within nodes of standing waves	Mainly IDTs on LiNbO_3,_ PDMS channels or Petri dishes	Microparticles, red blood cells, *Escherichia coli, Caenorhabditis elegans*	[29,30,52,53]
Acoustic vortices and streaming forces to trap objects	IDTs of different shape and configuration on LiNbO_3_, PDMS channels or Petri dishes	Microparticles, microbubbles, exosomes	[53,54]
Acoustic tweezers to move objects along defined trajectories	IDTs of different shape and configuration on LiNbO_3_, PDMS channels or Petri dishes	Microparticles, breast cancer cells, zebrafish embryos	[[55], [56], [57], [58]]
Manipulation in 2D and assembly of multiple objects			
Concept	Device	Particle/cell type	References
Spheroid formation and cell aggregation	Mainly IDTs on LiNbO_3,_ PDMS channels or Petri dishes ZnO/Si piezoelements	Microparticles, yeast cells, tumor spheroids, neurospheroids	[33,[62], [63], [64], [65],^69^]
Spatial positioning of cells and co-culture systems	Mainly IDTs on LiNbO_3_, PDMS channels	HMVEC-d, HeLa, epithelial cells, fibroblasts, Schwann cells, DRG	[[66], [67], [68]]
Cell patterning within hydrogels	Mainly IDTs on LiNbO_3_, PDMS channels or open-top chambers	hASC, PC12, HUVEC, hMSC, NRVC, iPSC-CM, C2C12, neuroprogenitor cells	[27,28,31,45,48,[70], [71], [72], [73], [74], [75]]
Assembly in 2.5D or 3D and acoustic patterning of building blocks			
Concept	Particle/cell type	Outcome	References
Spheroid assembly into engineered constructs	HUVEC and hMSC	Perfusable microvasculature	[45]
Multicellular assembly into 3D structures	Fibroblasts, HUVEC, hepatocytes	Liver organoids; cellu-robots	[80,81]
In situ cell–polymer biograft assembly	HeLa, MC3T3-E1, P12	Macroscopic fibers/building blocks for tissue engineering	[32]
Short-term acoustic manipulation—effect after seconds			
Concept	Particles/cells	Outcome	References
Cell seeding with acoustic waves	Primary osteoblast-like cells, yeast cells	Fast, homogeneous cell seeding	[82,83]
Acoustic stimulation of cells	Cortical neurons	Altered excitability and action potential	[84]
Acoustic waves for laboratory practice	Cryopreservation of hUCM-MSCs	Increased viability	[85]
Midterm acoustic manipulation—from seconds to minutes to trap and sort objects			
Concept	Device/strategy	Particles/cells	References
Object trapping and sorting	Acoustofluidics, mainly PDMS microchannels assembled on IDTs and LiNbO_3_	Microparticles, nanoparticles, spheroids	[37,86,87,90]
Acoustofluidics for diagnostics	Mainly PDMS microchannels assembled on IDTs and LiNbO_3_	Red blood cells, lymphocytes, circulating tumor cells	[88,89,91]
Biomaterial design by acoustic waves/Chemical reactions	Topographical structuring of polymer precursors or extracellular matrix proteins	PEGDA or PDMS beads, O_2_ and CO_2_ as reactive agents, collagen, fibrinogen	[[92], [93], [94], [95], [96], [97]]
Long-term acoustic manipulation—the effect of sound waves on cell fate			
Concept	Particles/cells	Outcome	References
Cell viability after prolonged acoustic stimulation	HeLa, human B cells	Confirmed cell viability	[99,100]
Cell differentiation under acoustic stimulation	hASC	Chondrogenesis, osteogenesis	[[101], [102], [103]]
Effect of acoustic waves on microorganisms	*Escherichia coli;* *Pseudomonas aeruginosa*	Biofilm formation; susceptibility to antibiotics	[34,110]

Abbreviations: hUCM-MSC: human umbilical cord matrix mesenchymal stem cells; PDMS: polydimethylsiloxan; IDT: interdigital transducers; PEGDA: poly(ethylene glycol)di-acryloyl; HeLa: Henriette Lacks cervical cancer cell line; hASC: human adipose tissue-derived stem cells; hMSC: human mesenchymal stem cells; HMVEC-d: human dermal vascular endothelial cells; DRG: dorsal route ganglion (neurons); PC12: cell line of neuroblasts and eosinophilic cells; HUVEC: human umbilical vein endothelial cells; NRVC: neonatal rat ventricular cardiomyocytes; iPSC-CM: induced pluripotent stem cell-derived cardiomyocytes; C2C12: murine skeletal myoblasts.

The literature research was completed in summer and fall 2020 and winter 2021 using the following databases: Web of Science, google scholar, and PubMed. The literature was searched using the following terms: faraday waves, acoustic waves, standing waves, surface acoustic waves, sound-induced cell patterning, acoustic manipulation, and related terms. The discussed literature was then selected based on their focus on biological application.

## 2 Theoretical considerations and definition of terms

### 2.1 Classification of acoustic manipulation

Waves and resulting patterns are ubiquitously observed phenomena with a long-lasting history both on the experimental and theoretical levels on fluid dynamics. They can be observed in daily life: a coffee, gently shaken in a holder by the moving car, or the sand pattern induced by the sea waves. Such patterns occur under forces applied by standing waves. Standing waves are generated when a wave traveling in ​one direction is interacting with a wave traveling in the opposite direction. The overlapping waves generate a standing wave, which oscillates in time, but does not move in space. Points where the absolute amplitude is zero are called nodes, whereas the amplitude maxima are called antinodes. Such waves can exert forces on suspended particles and drive them toward ​the nodes or antinodes. This is essentially the reason why acoustic waves can be used to arrange particles or cells in space. The final effect and resulting particle distribution might be hardly distinguishable but underlying physical principles to induce the standing wave can be fundamentally different and are represented by the different setups used for particle displacement. Within the field of acoustic manipulation, some of the terms are used interchangeably and the physical definition is not always fully respected. In view of this, and for simplicity within this review, the different approaches can be roughly classified into the following three categories:

(a) Ultrasound manipulation by use of (standing) surface acoustic waves (SSAW or SAW) within a closed (micro)channel
(b) Ultrasound manipulation by use of bulk acoustic waves (BAWs) within a macroscaled container
(c) Low-frequency manipulation by use of Faraday waves within a macroscaled open container

In (a), a piezoelectric element, most commonly lithium niobate (LiNbO_3_), forms the vibrating element. Interdigital transducers (IDTs) will be addressed by frequency generators and transduce the signal to the piezoelement where an SAW is formed. By use of either a reflector or a second IDT that induces a surface wave traveling in opposite direction, an SSAW is formed in the solid crystal. Via a coupling liquid, a thin layer of oil or water, the pressure fluctuations of the piezoelement will be transferred to the (cell culture) medium within a microfluidic chamber. In there, acoustic radiation forces will drive particles or cells to the nodes. The applied frequencies are usually between 100 ​kHz and 10 ​MHz. These SAWs can either be created by one source and a reflector or two sources. Setups were used in different configurations with two or more IDTs. Different patterns were achieved by placing the IDTs perpendicular or rectangular to each other or by use of four IDTs that generate two standing waves that interact with each other. Devices based on these configurations are particularly interesting to be integrated within microfluidic devices and laboratory on a chip approaches, whereas they lack certain features to be used for manipulation at the macroscopic scale [26]. Ultrasound waves are prone to attenuation within liquid, and therefore experience changes in intensity over traveling distance.

In the second case (b), a piezoceramic transducer, most widely used is lead zirconate titanate (PZT), is electronically addressed to generate a bulk wave within a culture vessel, for example, a petri dish. The waves are transmitted through the liquid or hydrogel, reaching a reflector, for example, a glass or polymer lid. The traveling wave is reflected and generates a standing wave, or so-called BAW. The applied frequencies usually range from a few hundred kHz to 40 ​MHz. BAWs are parallel to the ultrasound-generating transducer and were, depending on the device configuration, applied vertically or horizontally [27,28]. Owing to the vertical cell distribution, Bouyer and colleagues were also referring to bioacoustic levitation instead of BAW in their work.

In setting (c), a frequency generator induces vertical vibration of a chamber filled with a liquid. At the liquid–air interface, so-called Faraday waves are formed. The periodic deformation of the air–liquid interface induces a hydrodynamic drag force, which acts on the particles or cells and moves them toward ​the minimum force, which is in the nodes and antinodes of the standing wave, depending on the particle size, density, acoustic refraction, and other properties. At high wave amplitudes, the wave field is disordered, which leads to turbulence ​and mixing of particles within the fluid system and/or carrier matrix and loss of the intended patterning. Faraday waves are created at low frequency, usually in the range of 40–200 ​Hz.

**Fig. 2:**
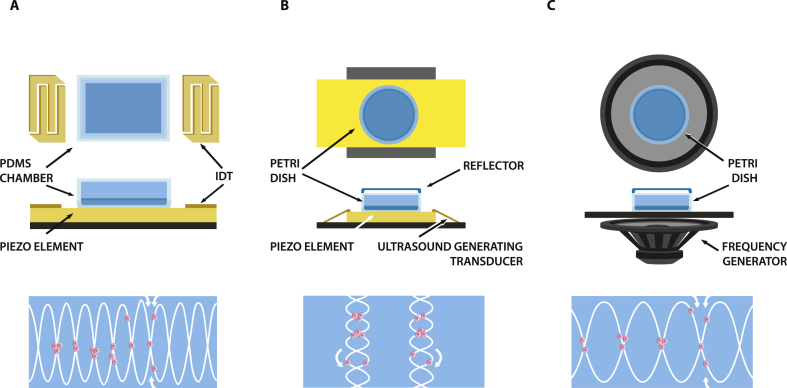
Schematic of the three main setups used for acoustic manipulation. (A) By use of ultrasound (100 ​kHz and 10 ​MHz), a piezoelectric element, most often consisting of LiNbO_3_, is addressed via interdigital transducers (IDT), inducing SAWs in a microchannel (polydimethylsiloxane chamber [PDMS]). (B) BAWs are induced by ultrasound, transduced to a ceramic piezo element (e.g. ZnO/Si). A reflector, covering the petri dish or container induces an opposing wave that overlaps with the incoming wave resulting in a vertical standing wave. (C) Low frequencies (40–200 ​Hz) are applied by a frequency generator to an open-top container or Petri dish that is placed on the generator. Faraday waves are formed at the liquid–air interface. In each scenario, cells (illustrated in pink) are moved toward ​the wave nodes by acoustic radiation or hydrodynamic forces.

The main differences among the three settings, in addition to the different technical features, are that in (a), the sample size is restricted to microchannels, whereas in (b) and (c), larger areas can be patterned and the use of standard labware (Petri dishes) is encouraged. Shi [29] and Ding [30] independently state that SAWs are superior to BAWs for certain applications, because of the smaller dimensions needed and hence the possibility for device miniaturization, yet also the higher resolution that can possibly be achieved by the higher frequency. In contrast, to pattern cells within hydrogels or more viscous liquids, BAWs are the preferred option [27]. Combining the advantages of both, Cohen used SAWs and BAWs to induce different patterns of linear arrangements or concentric rings, respectively [31]. Low-frequency manipulation by use of Faraday waves has the advantage that patterns can be achieved in larger culture wells without being susceptible to wave attenuation in fluid and that no increase in temperature is observed upon manipulation. Furthermore, pattern geometry can be adjusted by changing the container shape or material, which adds an additional degree of freedom to engineer custom-tailored cellular arrangements. The three principles are illustrated in Fig. 2.

The main factors influencing particle arrangement are parameters characteristic to the device (shape and dimensions, acoustic reflectance), to the liquid (compressibility, viscosity, density), to the particles (size, compressibility, density, rigidity, morphology, impedance, wettability), as well as the applied frequency, amplitude, and duration [27,32]. In an ultrasound approach of BAW, the applied sound waves and the generated forces must overcome the resistivity forces of particles within the fluid. In a simplified approach, this can be described by the following equations (1), (2) [27]:

(1) $$F_{resistive}=3\pi \eta dv$$

(2) $$F_{radiation}=\;\left(\frac{\pi }{24}\right)\left(k_{b}-k_{c}\right)d^{3}\left(\frac{2\pi }{\lambda }\right)P_{0}^{2}\;\sin\left(\frac{4\pi x}{\lambda }\right)$$

where in (1), η is the dynamic viscosity of the fluid and v is the velocity of the cell. In (2), *k*_*b*_ and *k*_*c*_ are the compressibility of the biomaterial (media/fluid precursor of the hydrogel) and the cell, respectively, *d* is the cell diameter, *P*_0_ is the pressure amplitude, and λ the wavelength. It becomes obvious ​that *F*_*radiation*_ must be larger in media/hydrogels of higher viscosity to move cells. This can be achieved by increasing the amplitude pressure. However, increasing the frequency would result in cell patterns/lines at a narrower distance, following equation (3):

(3) $$L=\;\frac{nc\;}{2f}=\frac{n\lambda }{2}$$

where *f* is the applied frequency, *n* the number of wave nodes, c the velocity, and *L* the length of the container.

Based on the above equations, where the resistive force is proportional to the diameter of the particles, and the radiation force scales with the volume of the particles (d^3^), it can be further concluded that the larger the particles, the higher the velocity based on the higher net force [33].

With respect to Faraday waves, the culture well depth is of importance, and the dispersion relation is described by the following equation (4) [34]:

(4) $$\omega^{2}=\;\left(gk+\;\frac{\sigma }{\rho }k^{3}\right)\tanh\left(kh\right)$$

where *ω* is the wave frequency, *h* is the height of the chamber/liquid, *k* is the wavenumber $\left(k=\frac{2\pi }{\lambda }\right)$, *σ* is the surface tension, *g* is the acceleration of gravity, and *ρ* the liquid density.

In addition to the radiation force that drives particles or cells toward ​the wave nodes, acoustic streaming is an important phenomenon to consider during acoustic patterning. Acoustic streaming describes a steady flow of particles within a fluid that can be observed as continuous movement through the medium rather than an assembly in defined positions. The streaming strongly depends on the applied frequency and amplitude, as well as the particle size and fluid depth [16,35]. Specifically, acoustic streaming can be reduced by diminishing the chamber height [36] and scales negatively with the particle size [37]. Assessing and quantifying the acoustic streaming effects is complicated by the many interdependent parameters and we would like to refer to an interesting paper on modeling acoustic streaming and radiation by Hahn and colleagues [38] and good descriptions and visualization of streaming effects by Ma and colleagues and Ding and colleagues, respectively [26,39]. For a good overview of the technical details in acoustic assembly, we would like to refer the reader to the review manuscripts highlighted earlier, in particular to Destgeer et al. and Mohanty et al. that provide ​excellent figures and schemes that help to understand the different scenarios [18,25].

### 2.2 In silico models of the Faraday instability

The formation of standing waves is dictated by various parameters, including frequency and amplitude of the vibrational force, volume and viscosity of the liquid, and properties of the particles such as, for example, size, density, or compressibility. Achieving the desired pattern is thus usually accompanied by optimizing interdependent variables in time-consuming trial and error experiments. Mathematical tools combined with computational power can support the researcher to achieve the system's configuration for the correct experimental design by predicting the outcome of different parameters and validating the results. Although Faraday waves have been investigated since decades, the first two-dimensional (2D) simulation of this phenomenon was only performed in the aughts of this century [40] and the first 3D simulation was computed in 2009 [41]. Importantly, the nodal patterns formed at the liquid–air interface by Faraday waves are not always indicative of the resulting particle arrangement. In addition to the created configuration of overlapping waves, the fluid's height plays a critical role (see Equation (4)), strongly influencing the outcome [42]. Throughout the fluid height, different hydrodynamic regimes were observed. For example, the flow in the region close to the bottom of the container generates oscillating boundary layers, which were subject of investigation by Wright et al. [43]. They created a particle ​transport simulation to prove the theory of oscillating boundary layers and how they are related to the thickness of the fluid. In summary, increasing the fluid thickness reduces the particle accumulation at the nodal location and the patterning capabilities are consequently reduced as well. This is an important aspect to consider when defining chamber thickness, choosing the diameter of particles and the applied amplitude. The strong influence of the z dimension in addition to the x–y plane led Périnet et al. [41] to create a 3D simulation of the Faraday instability. Their model could simulate the motion at the interface between two fluids undergoing Faraday waves. The authors argued that instead of modeling the curvature of the interface, it would be more accurate to model the capillary force acting on it when vertical stimulation on the container is applied. While taking into account the third dimension and additional complexity, it is relevant to also point out the computational time required for this type of simulation, which was 42 ​h for 1 ​s of physical time. Although this approach is more representative of the reality, researchers tend to use simpler 2D models, which are less complex and require less computational power and time. An example of 2D simulation of Faraday wave patterns can be found in the work of Chen et al. [44], where they modeled the interaction of the surface modes applying the 2D Swift Hohenberg type equation. In their simulation, the authors also included 200 ​μm polystyrene divinylbenzene particles within the fluid stimulated by Faraday instability. This simulation method is useful to visualize the Faraday instability pattern, but it requires the mathematical expression for the fluid surface deformation as input data. Thus, the pattern simulations are limited to square, stripes, or crystalline waveforms. As such, it is not possible to generalize the model prediction to other chamber shapes. In this study, MATLAB (from MathWorks) was used to solve the equations, plot the energy drift corresponding to each waveform and consequently illustrate the particles’ pattern. Petta et al. [45] visualized their pattern formation in MATLAB with a similar in silico approach, which arranged the particles in the desired final patterns. Furthermore, they predicted the trajectory followed by the dispersed tricalcium phosphate particles according to a Verlet algorithm, simulating particles interaction with the bottom of the chamber and the surrounding viscous fluid (gelatine methacryloyl [GelMA]). More complex arrangement of particles in fluids has been modeled focusing on the 3D configuration of periodic particle organization, as shown in the work of Guevara Vasquez et al. [46]. The authors used MATLAB to predict the arrangement of small particles driven by ultrasound applied to a curable resin to create crystalline-like material. They used the symmetry of the shapes of the vibration modes to investigate whether the global minimum of the radiation potential would be a point, a line, or a plane. With this simulation, it was predicted which geometry of particle organization, known as Bravais lattices, could be obtained by periodic waves. Most of the past simulations have considered the particle's dimension as several folds smaller compared to the wavelength. Therefore, Silva et al. [47] created an innovative model, which could evaluate the radiation force in a Faraday instability, acting on a particle with dimensions comparable to the wavelength. The envisioned application of their model was the design of a microfluidic acoustic trap. A plot of the radiation force map was used to show where the force was equal to zero, and hence where the trap well would take place. The authors used their model to calculate the theoretical maximum dimension of particles, which could be patterned in the acoustofluidics device, displacing one cell/particle per well trap. Modeling this phenomenon proved successful in determining whether the trapping position can be in the pressure nodes, antinodes, or midpoints, although the prediction of where the particle would be positioned depends on the mechanical properties of the particle and of the surrounding fluid. Therefore, the results in the experimental conditions can diverge from the in-silico results if the material properties set in the simulation are reasonably different from the experimental one. Often, researchers are interested in simulating the effect of several changing parameters (Multiphysics), closely resembling the real condition of a specific device where mechanical stresses and fluid dynamics are coexisting. Naseer et al. [48] simulated a microfluidic device where cells were patterned in GelMA under piezoelectric stimulation. The authors created a finite element method (FEM) analysis in COMSOL Multiphysics (from Comsol group) where they simulated the acoustic force patterning. The acoustic radiation force generated by the SAW was directed onto the cells inducing their movement. Using the theoretical findings of Settnes et al. [49], the authors could define the acoustic radiation force as a gradient of the potential energy acting on the cell. The model required to define the cell as a small object with precise radius, density, and compressibility values. The fluid, however, was defined as viscid and stimulated by a standing wave. In this approach, the resonance frequency of the device using the CFD eigenfrequency module in COMSOL was first evaluated, followed by modeling the acoustic pressure field with a frequency domain approach, and subsequently, the particle-tracing module to simulate how the pressure field would move the cells in GelMA was applied. Based on the simulations, the authors could optimize the physical dimensions of the device and the piezoelectric stimuli parameters. System complexity increased in the work of Collins et al. [50]. They built a model where patterns of particles inside a microfluidic channel were created by diffraction of ultrasound (generated from a piezoelectric element) on geometric features inserted inside the channel. From the results, they could predict the pattern periodicity in correlation with the surface curvature of the features in the channel. Furthermore, in a recent study by Ma et al. [39], non-symmetric patterns were created, which cannot be achieved from a standard acoustic field of a resonator. To do so, they modulated the homogeneous acoustic field generated from a transducer placing a 3D printed hologram, which reproduced the pattern shape, between the transducer and cell suspension. For the proper design of this system, the authors recurred to the acoustic–solid interaction and laminar flow modules of COMSOL Multiphysics, which were combined with FEM analysis to simulate how the acoustic field would be modulated into the desired shape by the 3D printed hologram. The Gor’kov potential distribution, the radiation force, and the streaming force generated within a liquid media were then simulated. The results showed the acoustic pressure distribution on the surface of the liquid, which resembled the designed 3D printed hologram. The complexity was reduced by assuming that the acoustic waves emanating from the source were symmetric along the z-axis and therefore only a cross-section of the beam in the z-direction was considered in the simulation. When applied to a cell suspension, cells were assembled according to the hologram. In conclusion, simulating specific applications requires the analysis of boundary conditions, the definition of the theoretical assumption which best fits the experimental condition, and the choice of the right computational tool. Thereby, experimental time can be reduced and complemented by developing an in silico model side by side to the in vitro model.

## 3 Acoustic patterning on different length scales

### 3.1 Single object manipulation and assembly in 1D

Simplified, acoustic tweezers take advantage of the forces acting on objects that can be trapped in the nodes of standing waves. By changing the frequency, the trapped object can be displaced-like tweezers grasping an object. Initial experimental work focused on trapping polymer particles or beads, but were later translated to the work with single cells [29]. By changing from a linear to an orthogonal IDT arrangement, fluorescent polystyrene beads, red blood cells, or *Escherichia coli* could be arranged in lines or aggregates, respectively. With a duration of only 10 ​s and a power input of 2,000 ​W/m^2^, the manipulation was relatively fast and of low energy. Later, Ding and colleagues [30] presented fascinating work on the manipulation of whole organisms. Their setup consisted of a microfluidic chamber, mounted onto LiNbO_3_ and four IDTs that were orthogonally placed around the chamber. The large bandwidth of the IDT defines the broad spectrum of nodes that can be induced, and thus free manipulation of objects in two dimensions. With this setup, the authors were the first to move a whole organism, *Caenorhabditis elegans,* in a contact-free, non-invasive manner at high precision. A power density of 0.5 ​nW/μm^2^ that is orders of magnitude lower than optical tweezers (∼10,000,000 less) clearly speaks for the friendliness of this method toward ​biological material. In addition to these proof-of-concept studies, Baresch and colleagues recently presented work on an in vitro model to demonstrate the use of acoustic tweezers for biomedical application [51]. By use of single-beam acoustical tweezers, microbubbles (with cargo) were trapped and manipulated through agarose or 2-cm-thick Tofu. Their unique approach of using a vortex trapping beam does not suffer from wavefront distortion (that is a change in the wave over distance, which leads to short-ranged effects) and can control microbubbles at high resolution. Advanced technical achievements were particularly brought forward by Prof. Huang and co-workers. In brief, conventional setups of IDTs on piezoelectric substrates were refined into dynamic and reconfigurable wave number–spiral acoustic tweezers by use of multiple circularly arranged IDTs [52]. Amplitudes and phases could be addressed independently, thereby allowing for precise manipulation of 10 ​μm particles by gradually controlled SAWs. Patterns could be transformed, merged, rotated, or translated along defined trajectories, which provide ​considerably improved flexibility over particle movement. The group further reported on user-friendly acoustic tweezers in standard Petri dishes that do not require microchannel fabrication [53]. Three different setups were developed that allowed (a) to pattern objects directly in a Petri dish, (b) to induce asymmetric waves using a tilted piezoelectric transducer to concentrate objects within the eye of a vortex, and (c) to use holographic IDTs underneath the Petri dish to locally stimulate or lyse cells and trap biomolecules. The idea of acoustic vortices to trap objects and/or concentrate analytes or nanoparticles was further developed into an acoustofluidic centrifuge [54]. A microfabricated polydimethylsiloxane chamber (PDMS) chamber was assembled on slanted IDTs. A phase shift in frequency can be used to generate two centrifuges within one chamber among which particles can be distributed. Thereby, subpopulations of exosomes could be separated as a model system, which provides tremendous potential for future application in, for example, point-of-care diagnostics. The latter work is a good example that acoustic tweezers can not only be considered as devices that trap objects within the wave nodes by acoustic radiation, but also take advantage of acoustic streaming forces. Following this concept and by use of specifically positioned microstructures within the PDMS microchannel, distinct ultrasonic streaming was achieved, which allowed for controled moving of objects along a defined path [55]. The same group further developed a simple, disposable device of cylindrical acoustic tweezers that make use of increased acoustic streaming to trap objects that are 1/400 of the incident wavelength [56]. The device bestows by its potential to manipulate subwavelength particles, which has not been achieved with conventional acoustic tweezers. A different project developed miniaturized acoustic tweezers by combining acoustic tweezers and holography to position and move individual cells [57]. Spiraling holographic transducers were excited and generated spherically focused acoustic vortices within a glass substrate that created a trap within a superimposed PDMS chamber. To demonstrate the power of this setup, a single breast cancer cell was picked up and moved in a slalom course among other cells. With a comparable rationale, Zhu et al. developed acoustohydrodynamic tweezers ​to perform in situ mixing, droplet transport along defined trajectories, and droplet merging [58]. By use of multi-unit arrays of piezoelectric elements and additional non-actuated elements, stable acoustic vortices were created. These allowed for dynamic control over objects by individual and alternating addressing. Oil in water droplets, polystyrene particles, and even zebrafish embryos were thereby moved along a defined path. Despite these exciting works, the effect of acoustic streaming on cell fate has often been neglected and is, apart from standard live/dead assays, seldomly addressed. As discussed by Guo et al., cell lysis in response to acoustic streaming was observed, and strategically positioned IDTs to reduce the streaming effect were suggested [23]. Furthermore, without any effect on cell viability, acoustic streaming was shown to influence cell attachment, cell spreading, and metabolic activity [59]. Human keratinocytes, mouse fibroblasts, human mesenchymal stem cells, and mouse osteosarcoma were subjected to acoustic streaming at 30–600 ​MHz and shown to respond differently to the applied ultrasound. Acoustic stimulation of osteogenic cells (MC3T3-E1) at 3.3 ​MHz for 300 ​ms pulses at a frequency of 0.5 ​Hz over prolonged time resulted in changes in the actin skeleton rearrangement, which was also attributed to the fluid shear stress induced by acoustic streaming [60]. These results underpin the importance of assessing specific cell responses after acoustic manipulation that might be overseen in conventional live/dead assays. This work is one of the few that investigated such questions and we believe that more research is needed to elucidate the effect of acoustic manipulation on cell fate. In a very elegant way, Shapiro and colleagues could bypass some of the boundaries related to fluid shear forces during acoustic patterning and material effects in general by a new approach with sonolithography [61]. Different from patterning in carrier matrices, particles in the air will have significantly different density and compressibility to their surroundings. Thus, a nebulizer was used to bring particles into an ultrasonic field that induced a patterning in air, before deposition on a substrate. The method was successfully applied to water droplets, colored sand, and cells.

It will be very interesting to see the previously discussed new developments translating from proof of concepts into use in cell biology, tissue engineering or diagnostics and clinical application.

### 3.2 Manipulation in 2D and assembly of multiple objects

Moving further in terms of length scale, sound-induced manipulation was also used to aggregate multiple objects in x and y directions. In response to the applied frequency or amplitude, cells could be aggregated within one node, thereby forming spheroids or organoids. By combining SAW and BAW, Zhang et al. [62] addressed microparticles of 1 or 10 ​μm in diameter, respectively. In brief, SAWs were induced by IDTs placed left and right of a PDMS chamber mounted on LiNbO_3_, with an additional ceramic piezo element underneath the PDMS chamber that induced the BAW. Their setup allowed to have a system where 1 ​μm particles were ordered in lines and the 10 ​μm particles were trapped on a grid of points. This report is one of the few where two different waves allowed for a bimodal distribution within the same well and could open up possibilities for particle separation. To overcome certain drawbacks of brittleness, expensiveness, and not easy integration within microchannels that surround the use of LiNbO_3_, a ZnO/Si piezoelement was used by Tao and co-workers [33]. Thin-film piezoelectric devices based on ZnO or aluminum nitride ​can seamlessly be integrated within microchips, which is an advantage to conventional setups. Microchannel width, thickness, and frequency were changed to control the distance between patterned lines. At increasing frequency (and thus smaller distance between the nodes), decreasing line spaces of polystyrene particles (6 or 10 ​μm in diameter) or yeast cells were induced. The authors further observed that larger microparticles moved faster than small ones, which is in line with equations (2), (3)). Although there was no specific biological readout after yeast cell patterning, the authors could conclude that the method can be used for particles of different sizes, as well as cells, without compromising their viability.

A fast and easy method to generate spheroids used acoustofluidics and a channel with spacers functionalized with a protein-repellent amphiphilic coating [63]. The method allowed for a standardized production of highly homogeneous spheroids. Furthermore, the device was coupled to an automatic analytical system of bright field microscopy, confocal microscopy, and flow cytometry. By this, the platform holds great potential for future high throughput production and analysis. Underpinning the necessity of advanced methods to produce spheroids at high throughput and reproducibility, Chen and colleagues focused on the formation of tumor spheroids using an acoustofluidic approach with a throughput of up to 12,000 spheroids per chamber [64]. A PDMS chamber with multiple outlets was mounted onto an LiNbO_3_ piezoelectric substrate. On applying SAW (3 ​min), cells in suspension assembled in the nodes. Spheroids were kept in the channels for about 2 ​h and then transferred to low adhesion plates for further culturing, resulting in a yield of 50% after this additional culture (6,000 spheroids after the entire procedure). Outstanding in this publication is the large number of cell types the authors tested (3 mouse cell lines, 3 human cell lines), clearly indicating the robustness of their approach for various tumor models. As with other spheroid formation techniques, the quality (viability, size) of the spheroid largely depends on the cell type used. At the expense of lower throughput, yet the clear advantage of working in standard 24-well plates, Kurashina and colleagues formed spheroids of BT-474 human breast cancer cells [65]. By use of high frequency (MHz) BAW, induced via a ceramic piezoelectric plate, BT-474 ​cells were fused into aggregates in the middle of the wells. Intermittent excitation was used to keep the temperature low and the final applied power was 75 ​mW per well. The authors see the strength of their method compared to conventional approaches to form spheroids, that it is less cumbersome and not only feasible for highly skilled technicians. The one-step procedure is less prone to contamination and overall simpler. Of note, however, the final results were preceded by an extensive parametric study, and only one set of parameters resulted in spheroid formation. It underpins the necessity of tightly adjusted systems with respect to well plate shape and material as well as applied frequency and duration. In a different approach to study cancer cell fate, co-culture systems of epithelial cancer cells (HeLa) and human dermal microvascular endothelial cells (HMVEC-d) were investigated by Li et al. [66]. A PDMS chamber was placed on top of an LiNbO_3_ piezoelectric substrate and addressed with IDTs. The IDTs were facing each other, thereby inducing line patterns of both cell types (at 12.78 ​MHz for 10 ​s). Co-culture systems were established by first patterning one cell type in media, followed by a phase shift of 180° to pattern the second cell type adjacent to the first one to generate alternating, parallel lines of HeLa and HMVEC-d cells. In the co-culture system, increased cancer cell migration was observed as opposed to HeLa cells only. The authors furthermore stress on the need to have devices and methods to achieve controlled co-culture and point out drawbacks of conventional methods, such as micropatterning, where cells might be influenced by the substrate heterogeneities, while bioprinting has relatively low patterning resolution. Furthermore, mechanical confinements might induce additional stress to the cells. With their system, a relatively small cell number in a volume of only 2 ​μL was used, which is particularly interesting if precious, patient-derived cells were used in the future. A different co-culture system with gentle manufacturing steps was achieved by superimposing a previously assembled layer of epithelial cells on a monolayer of fibroblasts [67]. In an acoustofluidic bioreactor, epithelial cells were assembled into sheet-like structures. Importantly, cell sheet contraction under levitation had to be inhibited by Ca^2+^-free culture media, but then provided a fast and gentle method for cell sheet production and subsequent assembly with fibroblasts.

The aforementioned studies were primarily addressing the assembly of objects or cells into spheroids and continuous culture in standard labware. For potential in vitro tissue engineering, however, the use of appropriate biomaterial matrices would be important and cell patterning is ideally accomplished in a suitable hydrogel precursor.

Chansoria and colleagues emphasized on this and presented work that uses BAW to pattern cells in alginate [27]. To evaluate their method, the authors performed a parametric study and changed frequency (0.71, 1, 1.5, or 2 ​MHz), signal voltage amplitude (100 or 200 ​mVpp), bioink viscosity (5, or 70 ​cP), and actuation duration (10 or 20 ​min). The resulting alignment, viability, and metabolic activity of human adipose-derived stem cells (hASCs) were assessed. Their motivation was to form anisotropic cell assemblies that resemble the parallel oriented cell structure within a variety of tissues such as tendon or muscle. In previous research, cell orientation along a parallel axis has primarily been induced by topographical cues, whereas in their work, cell aggregation could be achieved by remote, acoustic patterning. Their setup furthermore used an open-top chamber, rather than a closed microchannel, which would allow for additional use of bioprinters on top to create multilayered constructs. Moderate frequency and high amplitude reduced cell viability, interarray spacing increased at decreasing frequency, and the cell concentration within a line was proportional to the amplitude and patterning duration. A very recent study by Cohen et al. [31] used superimposed SAW and BAW and presented results on two different patterns. SAW was used to assemble cells into line patterns, whereas the latter one resulted in concentric rings. Neuron-like PC12 ​cells and primary dorsal root ganglia neurons were patterned on top of collagen hydrogels and kept in culture over 6 days without impaired viability. Over time, PC12 neuronal cells grew branches toward ​the other patterns and started to infiltrate the hydrogel. Ring size or line dimensions could be tuned by adjusting the applied frequency and/or the used cell concentration (1 ​× ​10^5^, 2.5 ​× ​10^5^, and 1 ​× ​10^6^ ​cells ​mL^−1^). Although without any in-depth biological evaluation, the study is very interesting in that it shows the many degrees of freedom with respect to obtained patterns, different devices, and distinct applied parameters. Neurite outgrowth was further assessed by placing dorsal route ganglion (DRG) on top of patterned lines of Schwann cells [68]. Briefly, a heptagonal acoustic tweezer device where the seven IDTs could be addressed individually and consecutively with a phase shift was used to generate dynamic cell patterns in a controlled fashion. Parallel lines or perpendicular tartan-like cell lines were generated that were subsequently used to study neurite outgrowth in response to the neighboring cell patterns. A more uniform and directed outgrowth was observed on patterned Schwann cells compared to randomly oriented. Another study on in vitro brain modeling focused on understanding Alzheimer's disease (AD) [69]. Four piezoelectric transducers were arranged around a 35 ​mm Petri dish and actuated at 1 ​MHz to produce BAWs. Thereby, neurospheroids and amyloid-beta plaques were assembled in 3D to simulate and investigate AD. In each approach, the ordered and hierarchical structure of the cell assembly and the freedom over the acquired geometry will help to mimic the natural situation more closely and develop a better understanding of neuronal disorders. Bouyer and co-workers [28] presented a single-step bioacoustics levitational strategy (BAL) to pattern human stem cell-derived neuroprogenitor cells (NPCs). The authors highlight the importance of in vitro brain engineering and present a method where cell patterns are formed by BAW and resulting radiation pressure in a fibrin gel. The device consisted of a ceramic transducer driven by a waveform generator, an acoustic resonant chamber, and a Plexiglas acoustic reflector. The system was first evaluated with fluorescent polystyrene beads of 8, 49, or 202 ​μm in diameter. By changing the frequency, the number of layers and the distance between the layers could be adjusted. Subsequently, NPCs were patterned within 2 ​s, but continuously stimulated for another 10 ​min during which the fibrin polymerized. In addition to cell viability assays, NPCs were cultured for additional 30 days and assessed for their neuronal differentiation. One of the earliest reports on vascular network formation induced by ultrasound comes from the Hocking group at Rochester University. In 2011, Garvin and colleagues [70] applied ultrasound to induce vascular network formation of human umbilical vein endothelial cells (HUVECs) within collagen gels. Patterned, densely condensed HUVECs were superior in angiogenesis, compared to non-patterned cells, indicated by increased sprouting. The authors emphasized the importance of dense cell packing, and that lack thereof would not result in vessel sprouting. After 4 days in culture, sprout length and thickness were quantified based on image analysis. Furthermore, the authors observed that the surrounding matrix, that is, collagen fibers, were aligned along the sprouting axis, in accordance with alignment and condensation observed in vivo. A follow-up study by the same group [71] investigated the influence of different parameters on HUVEC patterning and vascular morphogenesis. Within a collagen gel, HUVECs were aligned in parallel lines, with varying distances of 1,500, 750, or 375 ​μm between the cell-bands in response to the applied frequency of 0.5, 1, or 2 ​MHz, respectively. After patterning, HUVECs were cultured for an additional 10 days, leading to the conclusion that the initial patterning had a significant effect on all further downstream events, ranging from density, tube formation, inner tube diameter, and sprouting. Different from other studies, the patterns were characterized by following an analytical algorithm, taking into account the lateral distance between adjacent peaks or adjacent troughs to quantify the distance between nodal and antinodal regions, thereby providing a metric of the distance between adjacent planar cell bands. In addition, the full-width, half-maximum value of each peak was used to quantify the width of the patterned cell bands, followed by analyzing the differences in the magnitudes of peaks and adjacent troughs to quantify the node-to-anti-node contrast. This contrast allowed the researchers to quantify a parameter of relative differences in local cell concentration between nodal and antinodal regions. It is one of the few reports, where a thorough numerical characterization of the obtained patterns was accomplished. In a very recent study, Petta and colleagues [45] demonstrated the formation of vascular networks within a fibrin hydrogel after patterning co-cultures of HUVEC and hMSC at low frequencies below 100 ​Hz. The remote and gentle assembly of cells into highly condensed frameworks that mimic the physiological cell density was shown superior in generating perfusable vascular structures at lower initial cell number compared to traditional methods such as microfluidics. Kang and colleagues generated a vascular pattern that was successfully implanted in animals [72]. Anastomosis and revascularization could be established in a rat model of ischemic hind limb injury, resulting in recovered function after 28 days. To do so, HUVECs and hASCs were co-aligned in a catechol-conjugated hyaluronic acid hydrogel within a PDMS chamber by use of BAW. Depending on the chosen material of the reflector, intensity in z-direction could be adapted and the cell pattern within the hydrogel steered. Before the in vivo evaluation, cell-hydrogel constructs were cultured in vitro and thoroughly analyzed for secreted angiogenic cytokines and overall vasculature formation based on immunofluorescent staining.

In addition to vascularization, cardiac tissue engineering and cardiomyocyte patterning have been a prominent topic investigated by researchers [48,73]. Naseer and colleagues used LiNbO_3_ piezoelectric substrates that were addressed with slanted-finger interdigital transducers ​and induced cell patterning at high frequencies of 3.4, 4.6, or 6.4 ​MHz, respectively. Neonatal rat ventricular cardiomyocytes were manipulated in GelMA and crosslinked by UV after successful patterning. The cardiac beating was assessed by microscopy and quantified with an in-house built MATLAB code and preserved for 5–7 days in culture. By using low-frequency patterning in fibrin gels (127 ​Hz and 110 ​mV for 10 ​s, 5 times, with intervals of 10s), Serpooshan et al. [73] condensed human-induced pluripotent stem cell-derived cardiomyocytes (iPSC-CM) to match the high cell concentration and spatial organization that is observed in native tissue. Physiologically relevant cell condensation resulted in increased functionality compared to non-condensed cells. This was confirmed based on immunofluorescent staining of α-actinin and connexin 43 and tangential stress and video observations to assess the contractile forces. The team has certainly achieved a milestone in cardiac cell patterning and subsequent functional assessment, yet one drawback lies in the spatial distribution in the z-axis. After patterning, iSPC-CM were let to sediment to the bottom of the Petri dish, followed by gel-crosslinking. To this end, cell condensation was achieved in x–y direction, yet no 3D cardiac construct within a hydrogel could be achieved. Interestingly, when comparing both these studies, the often-discussed advantages, or disadvantages of using high or low frequency seems unimportant. The latter one will be beneficial for the design of cell-material constructs of clinically relevant size due to enhanced large-scale patterning at low frequency. Unfortunately, neither of the authors comment on increasing temperature or other side-effects during patterning, but cardiomyocyte functionality was maintained using both approaches. Differences in setup, cell type, and analytical methods certainly deny a direct comparison, but the parallels are obvious and encouraging for future users, that researchers with different setups can achieve comparable results and the choice of setup might not be determining the scientific success. Also using ultrasound, Armstrong and co-workers [74] patterned murine skeletal myoblasts (C2C12) into parallel lines at frequencies between 2 and 6 ​MHz. Outstanding in this work is that the versatility of the method was demonstrated in different hydrogels, using collagen, GelMA, agarose, Matrigel®, and poly(ethylene)-norbornene. After patterning, myotube formation and gene expression, as well as contractility were analyzed. Parallel alignment resulted in superior myotube maturation compared to non-patterned C2C12, further underlining the importance of high cell density for tissue formation. Having demonstrated the robustness of the method with different hydrogels—at least with the current model cell line—is encouraging for future research. The anisotropic orientation of C2C12 ​cells to form multinucleated myotubes was also induced by a combined bioprinting/acoustic excitation approach [75]. Cells were suspended in 5% GelMA and extruded with a bioprinter whose glass nozzle was modified with two piezoelectric plates. During extrusion, C2C12 were condensed and aligned within the bioink, resulting in increased myotube maturation in the final construct.

The previously discussed studies tremendously moved forward the field of acoustic manipulation, in particular, cell assembly and aggregation to mimic the high cell density found in nature. Geometry and shape of the patterns are dictated by the underlying wave structures and reach their limits given by the boundary conditions of well shape, applied frequency, viscosity, and properties of the used matrix. Unfortunately, in-depth characterization of materials used with respect to viscosities, viscoelastic properties, and gelation kinetics ​are often lacking and the different values were seldom reported in the literature. Despite the many different setups and applications, a lot of work is restricted to comparable or similar gelling formulations of GelMa [48,74,75], fibrin [28,45,73], collagen [31,39,70,71,74,76], or PEG-derivates [32,74,77]. Based on theoretical considerations by taking equation (1) into account, and systematic rheological characterization, it might be possible to define intrinsic formulation parameters at which patterning by acoustic waves is possible. This would open new possibilities for future material development and reduce the laborious iterative trial and error experimental approach.

To pattern custom-defined geometries, acoustic holograms are increasingly considered. The published articles are merely proof of concepts, showing bicycles, doves, or coin figures, but the underlying science is terrific and will without doubt increasingly gain attention in the future. Acoustic holograms with high spatial resolution were first introduced by Melde and colleagues in 2016 [78] and later applied to the patterning of polymeric particles by the same group [79]. PDMS particles were functionalized with UV-sensitive linkers, patterned within a hydrogel, and subsequently UV radiated to retain the pattern after switching off the ultrasound. A very recent study by the same group applied acoustic holograms for the spatial arrangement of cells [39]. Human colon cancer cells (HCT-116) were patterned by use of an acoustic hologram within a collagen matrix and assessed for viability after 1 week in culture. The fast patterning (2–3 ​min) resulting in clear shapes are very intriguing for further research. Numerical simulation that was accomplished by the authors could further support the design and understanding of patterns induced by acoustic holograms.

### 3.3 Assembly in 2.5D or 3D and acoustic patterning of building block

A recurring theme in biofabrication is the importance of hierarchy and the assembly of building blocks into more complex structures. Not surprisingly, acoustic manipulation has also reached out to the assembly of aggregated units to form constructs of either higher complexity or larger dimensions. One of the most extensive studies with respect to material and size of the used objects and resulting patterns was published by Chen et al. [44]. Poly(methyl methacrylate) (PMMA) chambers, varying in size and shape, were mounted onto a vibration generator to induce Faraday waves. The authors defined a new term for their work, called liquid-based-template, which is essentially sound-induced patterning in a liquid. GelMa, PEG, or PDMS beads ranging in size from 10 ​μm to 2 ​mm, fibroblasts (NIH 3T3 cells), cell spheroids, and cell-seeded microcarrier beads were arranged in an area of 100 ​mm^2^ to 10,000 ​mm^2^. In addition to a parameter study with a plethora of geometries, spheroids were used as building blocks and assembled further into larger cell aggregates. Work by the same authors further exploited the assembly of multicellular spheroids of heterogeneous cell population into larger structures, ultimately achieving the formation of a liver organoid [80]. Briefly, Faraday waves were explored to densely pack a variety of cells (frequency between 120 and 140 ​Hz). In a stepwise approach, co-culture assemblies of fibroblasts, GFP-HUVEC, and primary rat hepatocytes were prepared, followed by long-term cultures. In a first step, cell aggregates were formed in low adhesion plates and then transferred to the sound device to agglomerate further into larger objects. By this, a donut of densely packed cells was generated and assessed for functionality. A bile canaliculi network was formed after 6 days, emphasizing on the possibility to use this method for organoid formation. By use of a carrier matrix, Lata et al. [32] formed macroscopic assemblies of patterned cell-polymer fibers that are subsequently collocated into larger constructs. Their method is a promising way to manipulate individual cells or clusters of cells in a non-invasive, biocompatible, contactless, and label-free manner through SAW, formed by two opposing IDTs on an LiNbO_3_ substrate. Power intensity and frequency were set to 1.5 ​W ​cm^−2^ and 12.65 ​MHz, and ultimately applied to a tubular chamber where HeLa cells, MC3T3-E1 osteogenic precursor cells, or P12 neuronal cells in a PEGMA or GelMA hydrogel suspension flow through and are simultaneously crosslinked by UV. The cell-polymer fibers were then removed from the tubes and manually placed into shape for subsequent cell culture. With this setup, and cell patterning within a tube, the authors state to be the first to use SAW manipulation over a large working distance of ∼1 ​cm. This is particularly promising for the use of different culture well devices and the formation of larger tissue constructs. Following the idea of cellular assembly on the macroscopic level, Ren and co-workers published their work on cellu-robot formation based on acoustic cell patterning [81]. Their rationale was that macromolecular building blocks, and/or cells, could ultimately merge to form complex tissues in vitro. Fibroblasts (NIH-3T3 cells) were suspended in an alginate hydrogel and patterned into a ring. After 24–72 ​h in culture, alginate templates were dissolved, leaving behind a scaffold-free construct with cells in a ring shape. In the lid of 96-well plates by applying Faraday waves at the liquid–air interface, multiple donuts were then assembled into a bracelet. It was stated that the speed of assembly with Faraday waves is much faster than conventional methods, further motivating the use of sound for cellular assembly. The authors furthermore felt inspired by ‘*various geographical cooking styles*’ and assembled cell-donuts into shish kebabs.

**Section summary and discussion**

**Fig. 3:**
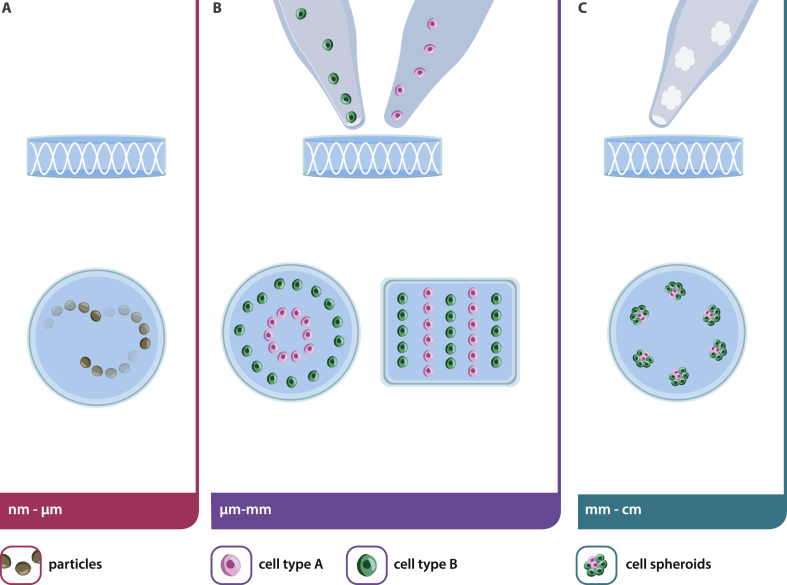
Schematic of acoustic manipulation on different length scales. (A) On the nm to μm scale, acoustic tweezers are used to trap particles or cells and position them in space. (B) On the μm to mm scale, particles or cells (or a combination thereof) are assembled in the x–y axis to form defined patterns. C) On the mm to cm scale, cell aggregates (spheroids or organoids) are assembled to form three-dimension in vitro engineered tissue constructs.

In summary, manipulation over different length scales focused on patterning beads and particles, the formation of organoids or spheroids in cell culture media, or within hydrogels (Fig. 3). The published work is clearly proof of (a) how different the devices are, (b) how incredibly heterogeneous the applied parameters, and (c) how imaginative and creative the research situated around acoustic manipulation is. In contrast to a large number of publications on the methods, numerical simulations, and proof of concept studies, in-depth biological evaluations are rather rare and have often been neglected. This is of course understandable and in the very nature of a step-by-step approach in the research community. To justify the use of acoustic manipulation for tissue engineering and morphogenesis, long-term studies ​and assessment of biological function are vital aspects that need addressing to understand the effect of sound waves on cell fate and potential tissue formation. Very interesting and promising for future applications is the large spectrum of cells that were patterned. Some of the earliest studies were performed with HUVEC and vascular sprouting as a readily available readout for functionality, cancer cells were then among the most prominent to establish spheroid models, followed by cardiac or muscle cells to proof the design of anisotropic tissues, and NPCs to develop in vitro brain tissues. In the next chapter, we will discuss some of the long-term cell studies and how acoustic manipulation was used at different time scales. Further studies are also needed to elucidate the effect of changing frequency or amplitude on cell viability. Correlations have been reported by many authors, without having investigated the reasons for cell death. Importantly, only few researchers addressed side-effects such as a rise in temperature that could potentially affect cell viability or high radiation forces that might induce cell wall rupture.

## 4 Acoustic patterning on different timescales

### 4.1 Short-term acoustic manipulation—effect after seconds

Generally speaking, acoustic manipulation and resulting patterning happen ​within seconds and continuous stimulation can induce further effects. Here, we are separating the published work according to the timescale of patterning, in particular with or without subsequent processing. On the timescale of seconds fall the papers that discuss cell seeding on scaffolds assisted by acoustic waves [82]. Infiltration of primary osteoblast-like cells into porous, foam-like poly(caprolactone) (PCL) scaffolds was increased at an applied frequency of 20 ​MHz and a used power of 380 ​mW. Different from static seeding with lengthy sedimentation times, seeding under acoustic stimulation was accomplished within 10 ​s. The advantages were homogeneous cell distribution within the scaffold without compromised cell viability. Furthermore, the osteogenic differentiation potential was maintained. The group has further expanded this research to the seeding of yeast cells into hydrophilic hydroxyapatite and PCL scaffolds [83]. In this study, the seeding process was live monitored by computer tomography, which allowed for in-depth analysis of the traveling wave within the scaffold and particle/cell distribution, which is highly interesting from a basic research point of view. Highly intriguing is very recent work by Clennell et al. on short-term acoustic stimulation of neurons [84]. Primary rat cortical neurons were stimulated for 40 ​s at 200 ​Hz and assessed for signal transduction by use of patch clamping. The acoustic stimulation changed the action potential kinetics and excitability for a duration of up to 8 ​h. Such results provide further evidence that the effect of acoustic manipulation is most likely much further reaching than what is conventionally assessed by viability assays and pattern characterization.

An unexpected application of acoustics was reported by Farooq et al. [85]. A standard laboratory on a chip SAW device based on four IDTs on LiNbO_3_ with a PDMS chamber was used to load and remove cryoprotective agents to and from cells. Within 1 ​min, the cryoprotective agent was removed, which resulted in significantly increased viability compared to conventional thawing and a reduced osmotic shock. Whether this method will find broad application for the cryopreservation of sensitive cells remains open and further research for increased throughput is needed.

### 4.2 Midterm acoustic manipulation—from seconds to minutes to trap and sort objects

As with many other research approaches in acoustic manipulation, early work started with separating particles and did not focus on biological objects [37,86]. Habibi and colleagues, for example [87], trapped microparticles in acoustic nodes of a microchannel and managed to thereby trap nanoparticles that were attracted by the microbeads. Acoustofluidics were also used to trap cells to perform single-cell analysis [88]. At high frequencies (>100 ​MHz), one cell per acoustic well was trapped and, different from mechanical trapping, cells are less likely to adhere to the channel they are trapped in. Their new technique, abbreviated as OCPM for one cell per acoustic well, is formed by IDTs that are arranged around a microchannel, which is placed on an LiNbO_3_ substrate. Multiple sizes of the microchannel were used to address objects of different dimensions in the micrometer range. Specifically, red blood cells and round lymphocytes were trapped and assessed for viability afterward. Red blood cells were also investigated by Richard et al. [89]. By use of an SU-8–based microchannel and SAW at an applied frequency of 12–13 ​MHz and 120–150 ​mW, the authors presented a proof-of-concept study on cell trapping. Work by Cai and colleagues [90] focused on the manipulation of spheroids. With their developed digital acoustofluidic method, spheroids of 20–300 ​μm in diameter could be trapped and positioned at high spatial resolution. These methods all have great potential to separate cells for analytical reasons, purify blood, or for filtration purposes. Ideally, devices allow for flow-through analysis at high throughput. With a clear clinical application in mind, acoustofluidics were also used to separate circulating tumor cells (CTCs) from blood cells in an in vitro approach [91]. An array of straight IDTs and focused IDTs were used to generate SAW and an asymmetric traveling pulsed SAW within a microchannel. CTCs were pushed away from the blood cells and streamed into a different channel, which allowed efficient separation with increasing cycling number.

Still on the same time scale, but following a different rationale, Mei et al. [92] and Piperno et al. [93] applied acoustic waves for a short time, yet with a long-term effect, namely assembly of polymer precursors into architecturally or topographically distinct features. In the first publication, PEGDA was UV-crosslinked within a chamber on an LiNbO_3_/IDT device. The applied waves resulted in a topographically structured polymer substrate. Of particular interest, the closed chamber approach could be interesting for future research to avoid oxygen-terminated radical polymerization. Piperno and colleagues followed a comparable rationale to in situ pattern and crosslink PDMS beads that were functionalized with a UV-sensitive linker. Thereby, patterning objects of an ongoing chemical reaction could be achieved, which opens up new avenues in scaffold fabrication. Following up on these studies, Wang et al. [77] used acoustic waves to pattern PEGDA that is then UV-crosslinked to retain the wavy pattern. Topographically distinct substrates are vital in research addressing contact guidance to align cells and the acoustic, one-step process can be an addition to conventional approaches with molds or additive manufacturing. On the downside, region selective patterning, and the possibility to produce more complex patterns, not only waves, is lacking. To overcome this issue, the authors used acoustic waveguides to produce locally defined patterns, but the experimental data and figures could not reproduce the large possible patterns they predicted numerically. This aside, it could be a step forward to produce topographically distinct patterns at high resolution and in a one-step process. Intriguing work on sound-triggered chemical reactions has recently been published by Hwang et al. [94]. The authors took advantage of redox systems that change their state in response to, for example, O_2_ or CO_2_ concentrations. Specifically_,_ chemical reactions were spatially controlled by applying low-frequency sound waves (40 ​Hz) that induced a patterned O_2_ or CO_2_ distribution and thereby locally triggered the reaction. In this proof-of-concept work, reactions with a visible color shift (the transition from methyl viologen radical to methyl viologen or use of bromothymol blue that changes color upon change in pH, induced by CO_2_ dissolution) were used, but the concept could in principle be translated to more biologically relevant reactions. Sound-induced segregation of chemical compounds could thus be envisaged as novel approaches to develop scaffolds with topographical features or chemical gradients. A different type of material templating, namely on collagen remodeling, was presented by the group around Dalecki and Hocking [95,96]. By use of ultrasound (7.8 or 8.8 ​MHz), density and fiber orientation of collagen within a hydrogel was increased locally, resulting in condensed collagen structures. Depending on the applied force, the spatial organization of collagen fibers was different, either radially distributed, or at higher power input, collagen condensation was at different spots spatially distributed. Adhesion and migration of fibroblasts, isolated from fibronectin-deficient mice, were then investigated, and shown to preferentially migrate toward ​or along the condensed collagen fibers. Ultrasound-induced mechanical forces could therefore remodel the ECM ​and specifically change cell adhesion or migration. It could also be further explored as scaffold templating to steer cell responses. In situ hydrogel gelation has also been reported by Nele and colleagues in a highly intriguing approach [97]. They explored the forces generated by ultrasound to burst liposomes or bubbles that then release cargo to trigger hydrogel polymerization. Specifically, Ca^2+^ was released, which activated enzymes that further triggered the polymerization or crosslinking of fibrinogen molecules. The method is highly versatile and could be applied to any ion-dependent enzyme and gelation system. The authors suggest this method to be used in various settings where UV-crosslinking would be hindered (for example through opaque materials), but have not reported on the feasibility of using this method in combination with cell-seeded hydrogels. It would be very interesting to further explore the method in combination with biological material.

### 4.3 Long-term acoustic manipulation—the effect of sound waves on cell fate

Moving objects or cells in space has been the primary focus of the previously discussed research efforts. Inherent to moving cells by acoustic waves are the exerted radiation forces, hydrodynamic forces, and shear forces, which might have an additional effect on cells. Researchers have thus exploited the response of cells that were subjected to acoustic waves over extended periods. This field of research is still in its infancy, but the remote control over cell differentiation or maturation will, without doubt, be the subject of future studies. To stimulate ECM production and maturation of spheroids, Sriphutkiat et al. [98] used acoustic patterning to first aggregate human liver carcinoma cells (HepG2). After patterning, cells were stimulated at 0.1 Watt for 30–90 ​min to induce ECM formation and aggregation into a stable spheroid, before sedimentation to the bottom of the well. After additional 7 days in culture, cell viability was confirmed. A standard setup of a PDMS cavity on an LiNbO_3_ piezoelectric device that was addressed via IDT ​was used. The experimental study was preceded by computational analysis to select appropriate parameters. Changes in frequency (in the MHz range) resulted in differences between speed of aggregation and size of the final spheroid. The authors have not accomplished any further biological analysis or studies with the generated spheroids, but raised an interesting point and suggested that spheroids can be used as components in bioink, hypothesizing that cell viability will be increasingly maintained compared to single-cell suspensions. With the main focus on computational models, Tani and co-workers [99] have also assessed the viability of HeLa cells that were subjected to ultrasound for 24 ​h. With a few limitations, the study provides some indication that ultrasound stimulation over multiple hours is not harmful to cells. In another proof-of-concept study, Vanherberghen et al. [100] could provide evidence that acoustic patterning for 12 ​h did not reduce cell viability of non-adherent human B cells compared to non-stimulated cells. Their research was motivated by the need of having a device that allows to trap and possibly sort cells, at simultaneous observation under the microscope—something that conventional fluorescent-activated flow cytometry or flow sorting is not capable of. Furthermore, their device consisting of a microplate platform with a single transducer and a silicon-glass microchip could simultaneously address wells in a 100-well plate, which is highly interesting for high throughput studies. Using a conventional IDT-LiNbO_3_ setup to induce SAW, Greco et al. [35] stimulated suspension growing monocyte cells (U-937) for 48 ​h at 50 ​MHz. Different from many other studies, the authors highlight the importance of studying side-effects (wanted or non-wanted) that acoustic waves might have. In particular, they suggested that the induced displacement of cells within the cell culture medium and resulting shear stresses experienced by the cells were responsible for the 30% increased proliferation of suspended monocytes compared to the static control. Shear stress is an important parameter that can have a beneficial effect on certain cell types, such as monocytes, that are experiencing flow conditions in their native environment, for example, within blood circulation. Another interesting point, if long-term studies are envisioned, is the increase in temperature that arises upon stimulation with ultrasound. In their experimental setup, the temperature was monitored by an IR camera in response to applied parameters. By this, the authors raised an important point that might have had a significant effect on reduced cell viability that has been reported in previous work upon ultrasound stimulation but was not reported or measured [88].

Actively inducing cell differentiation by applied acoustic waves has only been investigated by a limited number of researchers, particularly by Marycz and Marędziak [101,102]. Using low-frequency sound waves (25, 35, or 45 ​Hz), hASCs were stimulated over 21 days at 10 ​min stimulation each day and assessed for their chondrogenic, or osteogenic differentiation, respectively. At 25 ​Hz, most pronounced osteogenic differentiation was induced, indicated by, among others, increased calcium deposition, as well as osteocalcin and osteopontin expressions. Chondrogenic differentiation, however, was most pronounced at 35 ​Hz, evident by BMP-2 secretion and collagen II deposition, at reduced levels of collagen I deposition. Before acoustic manipulation was considered as a new field of research, pioneering work by Tirkkonen et al. [103] reported on enhanced osteogenic differentiation at reduced adipogenic differentiation of stem cells when hASCs were placed on a loudspeaker and stimulated at high magnitude and high frequency. Along this line, Halonen and colleagues investigated the effect of high-frequency vibrational stimulation on osteogenesis of human adipose stem cells [104].

Mechanical stimulation, or, for example, shock waves, have been known for their beneficial effect in various medical conditions to stimulate angiogenesis [105,106], in the field of cardiac regeneration [107,108] or to steer the inflammatory response [109]. Comparable investigations on sound-induced effects are currently lacking and need to be further investigated. The acting forces by applied acoustic waves might as well have a long-term effect on the phenotype. It will be very interesting to see where future studies will lead to and how acoustic manipulation can influence cell differentiation.

Long-term acoustic stimulation was also addressed in the field of microorganisms. Hong and colleagues investigated biofilm formation in response to applied Faraday waves [34]. The most pronounced biofilm formation of *E. coli* was located in the antinodes of the standing waves after 24 ​h. A new aspect raised by the authors that applies to long-term stimulation is the diffusion of nutrients and oxygen. Under stimulation, it is suggested that mixing of the fluid would increase oxygen transport and further enhance biofilm formation. However, under high applied amplitude and occurring turbulences, no biofilm formation was observed, and the molecules were mixed within the medium. Understanding bacterial growth within fluids under motion is of particular interest to control biofilm formation within tubular medical devices, for example, urinary catheters. This knowledge can also be used to stimulate biofilm formation in vitro, for example, for bacteria-derived cellulose production. In another study, SAW-treated biofilms were found to be more susceptible to antibiotics with successful biofilm eradication compared to the control groups where no stimulation was applied [110].

**Section summary and discussion**

**Fig. 4:**
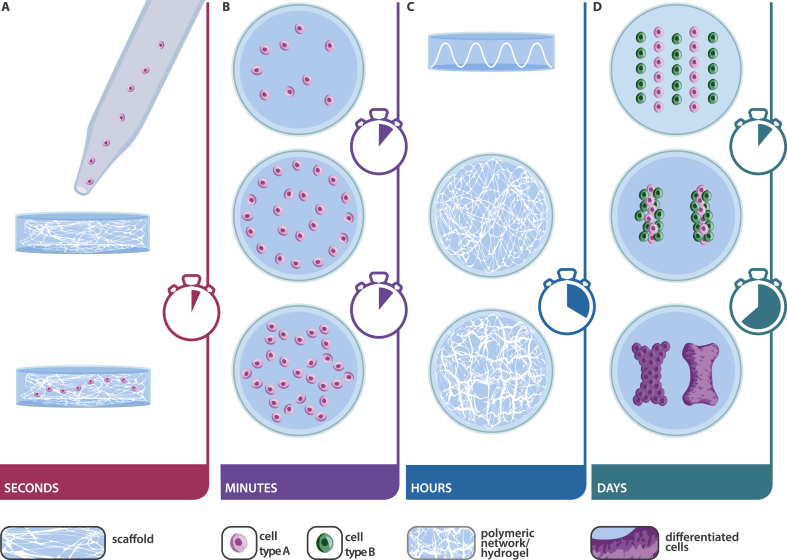
Schematic of acoustic manipulation on different time scales. (A) Within seconds, sound can assist in cell seeding within fibrous scaffolds. (B) Within seconds or minutes, cell assembly can be achieved by acoustic waves. (C) Over minutes or hours, acoustic manipulation can be leveraged to induce matrix remodeling of natural or synthetic scaffolds. (D) Cell differentiation can be promoted by acoustic manipulation over days or weeks.

Having been put into the scientific spotlight by acoustic tweezers, acoustic waves are capable and have been explored for far more than moving of single objects. This chapter provided some information on the broad effect sound can have and the explorative research ranging from particle or cell sorting ​to polymer substrate fabrication as well as cell differentiation (Fig. 4). The reviewed work presented concepts that can be useful for high throughput applications in pharmaceutical sciences as well as work on in vitro tissue engineering approaches. Still in its infancy, long-term effects of acoustic stimulation need to be further investigated, particularly to understand cell lineage commitment of stem cells and the potential of acoustic waves in regenerative medicine. As with many other techniques, individual parameters affecting cell fate are hard to be distinguished. It remains elusive, whether shear stress of moving cells within hydrogels or cell culture media are responsible for changes in proliferation or differentiation, or whether other mechanical components, temperature, or yet to be identified parameters, induce the observed effects. Most likely, synergistic combinations, at very specific thresholds can decide upon positive or negative effects and need to be investigated in extensive parameter studies. Changes in gene expression would be an interesting subject to study, and sound waves could be used to specifically alter the phenotype. In addition to considerations on the cellular level, gelation kinetics and mechanisms can significantly influence the success of long-term acoustic manipulation. Hydrogel crosslinking and concomitant cell entrapment will certainly alter the acting hydrodynamic forces and the resulting effect on cell fate.

## 5 Conclusion and outlook

Modern research developed new methods for cell manipulation at high spatial resolution, with great versatility and broad applicability. With joint forces of different disciplines, new machines have been created, often combining powerful techniques in the field of micro- and nanotechnology, complemented with modeling and computational approaches. Despite tremendous efforts and exciting results, we conclude that further research is needed to exploit the potential of these methods for regenerative medicine. In addition to manipulation on the single-cell level or formation of aggregates, generating functional tissues by use of sound waves is timely. Only very few reports that were discussed earlier performed in-depth characterization of the assembled cells and most of the analysis in the cited reports were restricted to a proof of concept and results on cell viability only. The number of long-term studies is very low, and it remains open, to which extent the acoustic manipulation has an effect on further downstream cell signaling and events. With respect to functional tissue constructs that can potentially restore or regenerate damaged organs upon implantation, vascularization is a major obstacle that needs addressing. Although a lot of studies have used endothelial cells and created vascular structures within hydrogels, the combination of different cell populations and the formation of truly 3D tissues is lacking to date. The enormous complexity of such hybrid tissue constructs requires elaborate experimental methods and design strategies to meet the clinical demands. In addition to implants of tissue-engineered constructs for regenerative medicine, creating in vitro models of patient-derived cells to either study diseases or evaluate new drug candidates and pharmaceutical approaches can be envisaged. Acoustic cell patterning opens new avenues to form multicellular structures and exploit the possibilities they offer.

Room for improvement can not only be found on the biological side, but also from a materials development point of view. Unfortunately, the large library of chemical reactions and polymers has only marginally been explored and new types of hydrogels should be designed that accommodate the appropriate properties for cell patterning. Specifically, viscosity during patterning, gelation kinetics, pore size, and overall mechanical properties should be addressed to meet the experimental protocol as well as the final application. Gelation kinetics, for example, either enzymatically driven, UV, pH, or temperature stimulated, have a major impact when long-term cell patterning is envisaged, and the viscosity of the fluid precursor needs to stay low throughout the pattern duration. A recent review by Spicer [111] provides good indications as to how hydrogels could potentially be designed to better meet the requirements postulated by cell biology to match the destination tissue. We suggest that even more interdisciplinary work is performed and that the challenges associated with sound-induced patterning are tackled by highly heterogeneous teams of scientists with varied backgrounds.

Another aspect we identified as being critical is the numerical quantification of achieved patterns or their quality control, respectively. Subjectively, one can easily distinguish patterned cells from homogeneously distributed ones, but quality control based on numerical values will be important for translation to the clinics. Comeau et al. [71] presented some interesting approaches on this matter and performed numerical analysis on their HUVEC patterns. An interesting study by Armstrong and colleagues further discussed this issue and presented an algorithm based on Voronoï Tessellation to study the distribution of cells [112]. This image-based analysis was used to find a correlation between the applied parameters and the quality of patterned cells. The authors suggest that this approach can be used in the future as a robust method for quality control of the devices, as well as statistical control of end results. Such evaluations and characterization are of paramount importance for standardization—not only for translational research but also for internal quality control in basic research.

The challenges ahead that need addressing are certainly situated around scaling up and the generation of material–cell constructs of clinically relevant size. Biofabricated tissues of high precision and reproducibility are needed, ideally accompanied by long-term culture for tissue maturation within 3D systems and bioreactor setups. To achieve this, mutual exchange among researchers must be encouraged and the community would benefit from broadly accessible knowledge, protocols, and device specifics or commercially available all-in-one systems. This would benefit researchers to increase reproducibility and comparability of results. Concluding, we see tremendous potential in acoustic manipulation and are curious to see how the field develops in the future.

## Author contributions

A.G.G.: conceptualisation, writing – original draft, N.D.M.: writing – original draft, D.E.: writing – review and editing, M.A.: writing – review and editing, T.S.: conceptualisation, writing - review and editing.

## Funding sources

The authors received funding from the 10.13039/100010661European Union’s Horizon 2020 Research and Innovation Program under grant agreement no. 860462 project PREMUROSA, the AO Research Institute Davos, the AO Development Incubator, and the AO CMF.

## Declaration of competing interest

The authors declare that they have no known competing financial interests or personal relationships that could have appeared to influence the work reported in this paper.
